# Clinical impact of ^99m^Tc-MAA SPECT/CT-based dosimetry in the radioembolization of liver malignancies with ^90^Y-loaded microspheres

**DOI:** 10.1007/s00259-015-3157-8

**Published:** 2015-09-04

**Authors:** Etienne Garin, Yan Rolland, Sophie Laffont, Julien Edeline

**Affiliations:** Department of Nuclear Medicine, Cancer Institute Eugène Marquis, CS 44229, F-35042 Rennes, France; University of Rennes 1, F-35043 Rennes, France; INSERM, U-991, Liver Metabolisms and Cancer, F-35033 Rennes, France; Department of Medical Imaging, Cancer Institute Eugène Marquis, CS 44229, F-35042 Rennes, France; Department of Medical Oncology, Cancer Institute Eugène Marquis, CS 44229, F-35042 Rennes, France

**Keywords:** Selective internal radiation therapy, Predictive dosimetry, Prognosis

## Abstract

Radioembolization with ^90^Y-loaded microspheres is increasingly used in the treatment of primary and secondary liver cancer. Technetium-99 m macroaggregated albumin (MAA) scintigraphy is used as a surrogate of microsphere distribution to assess lung or digestive shunting prior to therapy, based on tumoral targeting and dosimetry. To date, this has been the sole pre-therapeutic tool available for such evaluation. Several dosimetric approaches have been described using both glass and resin microspheres in hepatocellular carcinoma (HCC) and liver metastasis. Given that each product offers different specific activities and numbers of spheres injected, their radiobiological properties are believed to lightly differ. This paper summarizes and discusses the available studies focused on MAA-based dosimetry, particularly concentrating on potential confounding factors like clinical context, tumor size, cirrhosis, previous or concomitant therapy, and product used. In terms of the impact of tumoral dose in HCC, the results were concordant and a response relationship and tumoral threshold dose was clearly identified, especially in studies using glass microspheres. Tumoral dose has also been found to influence survival. The concept of treatment intensification has recently been introduced, yet despite several studies publishing interesting findings on the tumor dose-metastasis relationship, no consensus has been reached, and further clarification is thus required. Nor has the maximal tolerated dose to the liver been well documented, requiring more accurate evaluation. Lung dose was well described, despite recently identified factors influencing its evaluation, requiring further assessment. *Conclusion*: MAA SPECT/CT dosimetry is accurate in HCC and can now be used in order to achieve a fully customized approach, including treatment intensification. Yet further studies are warranted for the metastasis setting and evaluating the maximal tolerated liver dose.

## Introduction

Radioembolization with yttrium 90 (^90^Y)-loaded glass or resin microspheres is increasingly used in the treatment of primary and secondary liver cancers. In colorectal metastatic disease, its efficacy has been proven by two randomized studies [1, 2]. In hepatocellular carcinoma (HCC), the clinical results of this technique in large patient cohorts were encouraging, especially for those with portal vein thrombosis (PVT), though no randomized studies are currently available [3–7]. The efficacy of radioembolization in HCC patients with PVT had, however, already been demonstrated a long time ago using ^131^I-lipiodol [8], thus providing proof of the benefits of radioembolization in this context.

Simulation is always performed prior to injecting microspheres, consisting of two mandatory and complementary procedures: diagnostic angiography and ^99m^Tc macroaggregated albumin (MAA) scintigraphy. Diagnostic angiography is used to identify digestive arteries arising from the hepatic artery, with coil embolization when necessary, and to select the best catheter position for tumoral targeting. Cone-beam computed tomography (CT) is a valuable new procedure that can be performed during the angiography to aid in digestive vascularization recognition, as well as perfused tissue and tumoral targeting evaluation. While MAA scintigraphy was initially used solely for lung-shunt evaluation, it can also aid in digestive shunt recognition and dosimetric evaluation.

The goal of radioembolization is to deliver a tumoricidal absorbed dose to tumors while sparing the healthy liver tissue [9, 10]. To achieve the best efficacy and lowest toxicity profile, the tumor absorbed dose (TD) and that absorbed by healthy injected liver tissue (HILD) should be evaluated prior to commencing therapy.

From a radiobiological point of view, selective internal radiation therapy (SIRT) and external beam radiotherapy (EBRT) are critically different techniques [11]. This is primarily due to significant differences in dose distribution, which is heterogeneous in SIRT, dependent on the biodistribution of the therapeutic agent, and homogeneous in EBRT. The radiation exposure rate also varies between the techniques, with lower rates in SIRT compared to EBRT. The absorbed doses calculated in Gy for SIRT and EBRT are therefore not equivalent, with 1 Gy in EBRT not producing the same radiobiological disorders as 1 Gy in SIRT. A direct comparison of the value of absorbed doses between SIRT and EBRT is therefore impossible.

Nevertheless, only limited data is available concerning tumor dosimetry and the maximal tolerated healthy liver dose while using ^90^Y-loaded microspheres. Two kinds of dosimetric approaches can be used. Pre-therapeutic dosimetric evaluation can be performed using MAA single-photon emission CT (SPECT)/CT, presenting the major advantage of being available prior to ^90^Y-loaded microsphere injection, and also affording the possibility of directly influencing the treatment schedule. Post-therapeutic dosimetric evaluation, after ^90^Y-loaded microsphere injection, can be performed using either ^90^Y bremsstrahlung SPECT/CT or direct ^90^Y positron emission tomography (PET).

This paper sought to discuss the confounding factors that may have an impact on MAA dosimetry and the clinical dosimetric impact of MAA SPECT/CT-based dosimetry in radioembolization.

## Dosimetric tools and endpoints

### Evaluation of the physical absorbed dose

To date, several different dosimetric approaches have been described using both glass and resin microspheres in HCC and liver metastasis [12]. The main approaches seen are the classical medical internal radiation dose (MIRD) technique, Monte Carlo simulation, and kernel point evaluation. These provide the physically absorbed dose: D.

### The MIRD approach

The most widely used method is MIRD, assuming a homogeneous distribution of spheres.

As microspheres are not biodegradable and remain trapped in the vessels following initial embolization, the effective half-life is assumed to be equal to the physical half-life of yttrium 90.

The absorbed dose, D (in Gy), in an organ of mass, M (in Kg), with an organ activity, A (in GBq) of ^90^Y, is then calculated using the following simplified MIRD equation:

$$ {\mathrm{D}}_{\left(\mathrm{Gy}\right)}={\mathrm{A}}_{\left(\mathrm{GBq}\right)}.50/{\mathrm{M}}_{\left(\mathrm{K}\mathrm{g}\right)} $$

Doses can be calculated for different compartments, especially tumor, liver, healthy liver, and lung tissues.

Doses are calculated at the voxel level or expressed as the mean dose of a compartment.

The activity in each compartment, namely the injected liver, tumor, healthy injected liver, and lungs, is typically evaluated by means of MAA scintigraphy.

The compartment volumes, namely the injected liver, tumor, healthy injected liver, and non-injected liver, can be measured using CT or cone-beam CT.

More recently, it has been shown that volumes can be accurately measured using SPECT/CT. It is well known that SPECT alone cannot achieve accurate volume measurement, as it depends on the threshold used for the volume of interest (VOI) delineation, required for evaluating the structure of interest. A phantom study has, however, demonstrated that SPECT/CT volume measurement can be accurate if the thresholding is guided by an anatomical visualization of the VOI on the fusion images, producing a mean error rate <7 % [13].

Given the wide variations possible in hepatic vascularization in this context, the use of MAA SPECT/CT for volume measurement offers the advantage of providing a more functional evaluation of the volume that is truly perfused [13].

**Fig. 1:**
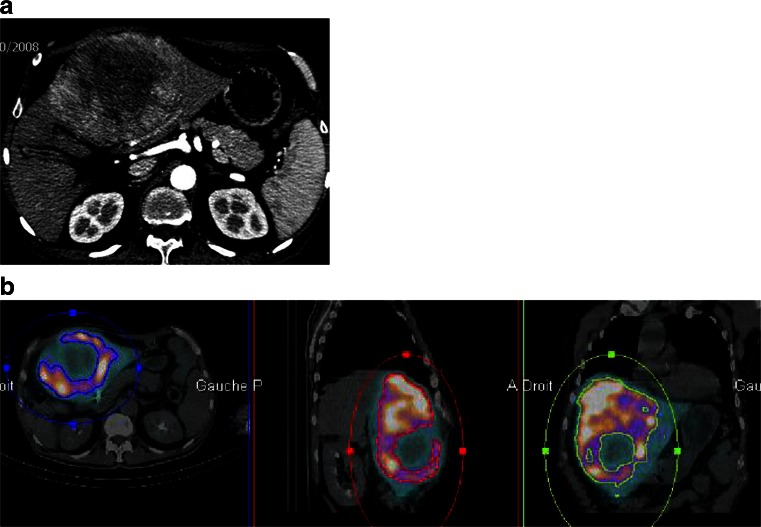
Tumoral VOI delineation using MAA SPECT/CT. **a**) CT slide: huge tumor of 12.1 × 17.4 × 9.7 cm with a large part of central necrosis. **b**) MAA/SPECT CT tumor volume segmentation. Based on MAA SPECT/CT, tumor volume is only 900 cc due to a large part of necrosis. Using CT segmentation, tumor volume is 1310 cc. Doing the hypothesis of absence of radioactivity uptake in necrosis, tumor dose based on MAA segmentation (excluding necrosis volume) is 1.45 fold higher than tumor dose based on CT segmentation (including necrosis volume)

We therefore conclude that two methods are available for tumor segmentation, offering significantly different results in terms of dosimetric evaluation. The first is morphological, with the tumor VOI delineated by means of CT, and then copied onto the SPECT imaging (or SPECT/CT) for count evaluation. In this situation, the tumor dose takes into account cold areas of necrosis. This method provides a relatively underestimated tumor dose. The second method, based only on SPECT (or SPECT/CT), is more functional. In this situation, only the hypervascularized part of the tumor is taken into account, with its dosimetry providing the basis of the tumor dosimetry, excluding tumor necrosis. This method provides a relatively overestimated tumor dose in comparison with the former morphological technique. An example of tumor segmentation with SPECT/CT is provided in Fig. 1.

### Drawbacks of the MIRD approach

MIRD does not, however, take into consideration the heterogeneity of dose distribution. It also disregards how the crossfire phenomenon affects the absorbed dose; namely, the irradiation of the tumor related to microspheres located in the surrounding healthy injected liver, and vice versa.

These two drawbacks can be overcome with the Monte Carlo simulation. Using a “Monte Carlo code,” this method accurately simulates the amount of energy deposed in tissue by a punctual source of radioactivity, considering both the random nature of particle interactions in tissue and tissue heterogeneity. However, its computing time is very long, up to several days, thus limiting its use.

The Dose-point Kernel method has been developed as a way of reducing this computing time. Rather than simulating energy deposition, this method only estimates the amount deposed in tissue by a punctual source of radioactivity, assuming a homogeneous tissue. This approach therefore takes into account the crossfire phenomenon, yet heterogeneity is still a problem, and the technique is consequently not routinely used.

We should not forget, however, that the above methods may be implemented in the future to further improve dosimetric evaluation, especially in difficult cases where MIRD may be insufficient.

Tumor size has a direct impact on SPECT/CT radioactivity quantification, namely the count of detected radioactive disintegration, carrying a risk of partial volume effect for lesions that are up to two times smaller than the spatial resolution of the gamma camera. This observation results in an underestimation of the quantification and thus of the calculated absorbed dose. The partial volume effect has been known to occur with lesions under 3 cm in size and constitutes a major risk for any smaller than 2 cm. This is why several teams exclude tumors smaller than 2 cm from dosimetric evaluation [12].

In cases of multifocal or infiltrative disease, tumor delineation may be difficult to perform or even impossible to achieve, and it may be impossible to accurately evaluate dosimetry, whichever method is used (MIRD, Monte Carlo or Kernel).

### Biologically effective dose calculation

The biologically effective dose (BED) can be implemented using the three principal dosimetric approaches mentioned above, namely MIRD, Monte Carlo, and kernel point. BEDs are based on correcting the physically absorbed dose D by means of radiobiological parameters. One of the advantages of this approach is that doses expressed as BED are usually comparable between all radiation therapy types, especially EBRT and SIRT.

BED is then calculated as below [14]:

$$ \mathrm{BED}=\mathrm{D}+{\mathrm{D}}^2\kern0.5em .\kern0.5em \left(\lambda /\ \left[\mu +\lambda \right]\right)\ .\ \left(1/\kern0.5em \left[\alpha /\upbeta \right]\right) $$

Parameters α and β are related to specific tissue parameters in terms of cell radiosensitivity, with α related to lethal damage and β to sublethal damage. The μ is linked to the repair of sublethal damage (μ = ln [2]/ T _½, repair_) and λ is the biologically effective decay constant. To date, the most widely used α/β ratios for SIRT are 10 and 2.5 Gy for tumor and normal liver tissue, respectively. The most widely used values for T_1/2,rep_ are 1.5 and 2.5 hours for tumor and normal liver tissue, respectively [14, 15].

Yet these parameters result from EBRT techniques with a specific irradiation configuration of dose, dose rate, and fractionation. New parameters must now be specifically defined for SIRT configurations, including the isotope and specific activity used, if we are to obtain a dosimetric evaluation that is comparable between each type of radiation therapy.

In the future, the use of BEDs could be of great interest, as we would be able to correct radiobiological parameters for several clinical parameters, like hypoxia, etc. Another outcome could be the possibility of implementing fully personalized definition parameters based on the patient’s biological status and tumor and liver genomic status.

### Dosimetric endpoints

The dosimetric endpoints vary depending on the product used, *i.e.,* resin or glass microspheres.

### Dosimetric endpoints for resin microspheres

Two rules governing activity definition are now available for resin microspheres: the body surface area method (BSA) and the partition model.

For the BSA method (results in m^2^), the activity to be injected (IA) is calculated using the following formulae:

For whole-liver injection:

$$ {\mathrm{IA}}_{\left(\mathrm{GBq}\right)}=\left(\mathrm{B}\mathrm{S}\mathrm{A}\hbox{--} 0.2\right)+\mathrm{fractional}\ \mathrm{tumor}\ \mathrm{involvement} $$

For lobar injection:

$$ {\mathrm{IA}}_{\left(\mathrm{GBq}\right)}=\left(\left[\mathrm{B}\mathrm{S}\mathrm{A}\ \hbox{--}\ 0.2\right]\right.+\mathrm{fractional}\ \mathrm{tumor}\ \mathrm{involvement}\left(\%\ \mathrm{of}\ \mathrm{treated}\ \mathrm{liver}\right) $$

The partition model, initially described by Ho et al. [16], has been declared the recommended choice, according to an expert panel, for HCC cases with delineable tumors [9]. This model consists in performing a dose evaluation of the tumor and healthy liver tissue. In the past, the tumor and healthy liver uptake were calculated using the ratio “r” of tumor uptake /non-tumor uptake, measured on planar MAA scintigraphy.

In current practice, tumor and non-tumor uptake are directly measured by SPECT or SPECT/CT.

Experts recommend a tumor dose (TD) of at least 120 Gy, whereas the dose to the healthy liver should not exceed 50-70 Gy, despite the lack of rigorous documentation of this limit [9]. In cases involving non-delineable tumors, the BSA method is used.

With regard to lung shunting fraction (LSF), the injected activity was adapted. According to SIRtex recommendations [17], ^90^Y-loaded resin microspheres are contra-indicated for cases exhibiting an LSF exceeding 20 %. The prescribed activity is reduced by 40 % for an LSF between 15 and 20 %, and by 20 % for one between 10 and 15 %. The full prescribed activity can be injected for an LSF <10 %.

Lung dose can also be directly calculated using the partition model for lung-dose estimation described by Ho et al. [16], using the following MIRD simplified formula:

$$ \mathrm{L}\ {\mathrm{D}}_{\left(\mathrm{Gy}\right)}={\mathrm{IA}}_{\left(\mathrm{GBq}\right)}.\ \mathrm{L}\mathrm{S}\mathrm{F}\ .\kern0.5em 50 $$

where IA is the injected activity of ^90^Y-loaded microspheres and LSF is the lung shunting fraction, and with the assumption of a uniform dose distribution, 1 kg lung mass, and that 1 GBq of ^90^Y delivers 50 Gy in a 1 kg mass.

A limit of 30 Gy is now more currently used in calculating lung dose, rather than the SIRtex recommendation regarding activity reduction based on LSF.

### Dosimetric endpoints and glass microspheres

With glass microspheres, the objective is to deliver a radiation dose of 120 ± 20 Gy to the injected liver volume (ILD), calculating the dose based on the accepted simplified MIRD formula given below [18]:

$$ \mathrm{I}\mathrm{L}\ {\mathrm{D}}_{\left(\mathrm{Gy}\right)}={\mathrm{IA}}_{\left(\mathrm{GBq}\right)}.\left(1-\mathrm{L}\mathrm{S}\mathrm{F}\right).\ 50/\ {\mathrm{M}}_{\left(\mathrm{K}\mathrm{g}\right)} $$

where IA represents the activity to be injected, LSF the lung shunting fraction, and M the mass of liver volume to be treated.

Lung dosimetry is also evaluated, assuming a lung mass of 1 kg, using the same Ho et al. simplified MIRD formula given in the previous paragraph.

The recommended maximal lung dose is 30 Gy for one treatment session and 50 Gy as a cumulative dose [18].

## MAA as a microsphere surrogate and confounding factors

### The physical properties of MAA and microspheres

MAA is made up of biodegradable particles, whose size are not well calibrated and are estimated to be between 10 to 150 μm. The majority, 90 %, measure between 10 and 40 μm and 1–2 % measure under 15 μm.

For microsphere simulation, between 150 and 250 MBq of ^99m^Tc-MAA are typically injected into the hepatic artery, along with approximately 0.5 × 10^6^ MAA particles [19]. As a comparison, we injected approximately 2 × 10^6^ spheres for 1.5 GBq of glass microspheres on Day 3 post-calibration, and around 30 × 10^6^ spheres for 1.5 GBq of resin microspheres.

Labeling proved to not be highly accurate, with the presence of ^99m^Tc possible. There is some uncertainty with regard to the stability of MAA after preparation, though no studies have thus far proven its instability. Labeling instability after injection has, however, already been reported [20], and the literature recommends performing the MAA scan as soon as possible after injection.

Despite their being approximately the same size, the specific activities and number of spheres injected differ between these two products. The mean size of glass microspheres is 25 ± 5 μm and their specific activity is 2500 Bq/sphere. For resin microspheres, the mean size is 32 ± 10 μm with a specific activity of only 50 Bq/sphere. Even if no comparative study is available, resin microspheres theoretically produce a stronger embolic effect due to the higher number of spheres injected for the same activity, and the discrepancies between MAA and resin microsphere distribution may be even more frequent.

On the other hand, Bilbao et al. reported contrasting findings when performing arterial injection of resin microspheres, which resulted in no microscopic signs of ischemia in healthy pig liver tissue following injection until stasis, with only 20 % of the vascularization classically arising from the hepatic artery [21]. Nevertheless, this observation using healthy tissue cannot be extrapolated to tumors, where 80 % of the vascularization classically arises from the hepatic artery, involving immature neovessels. The potential embolic and ischemic effect of resin microspheres in some tumors is still a matter of debate. This potential emphasizes the value of a slow injection of resin microspheres, accompanied by sequential angiographic monitoring in order to prevent stasis, which has been proven to be the primary cause for stopping delivery of resin microspheres before the full planned activity can be injected [22]. This embolic effect is thought to be stronger in small metastatic lesions [23], and probably produces an impact on therapeutic effectiveness. The potential embolic effect of resin microspheres thus only means that MAA-based dosimetric evaluation may be inaccurate in this context.

Due to this significant difference in specific activity and thus in the number of microspheres necessary for the same injected activity, the radiobiological properties of resin and glass microspheres are believed to differ [24, 25], requiring separate analysis [26]. A recent study based on microsphere distribution modeling clearly demonstrated that the different radiobiological effects of glass and resin microspheres were indeed related to differences in the specific activity and number of spheres injected [25].

### Confounding factors

Despite the absence of a specific study on this subject, it is more than likely that several confounding factors, other than microsphere type, may have an impact on the reproducibility between ^99m^Tc-MAA and microsphere distributions, including tumor type (e.g., primary or metastasis ), tumor vascularization, tumor size, prior therapy, MAA injection parameters, and angiographic considerations such as catheter position and vasoactive arterial status.

### Tumor type

In an HCC context, there are typically one or several large lesions that receive frequent first-line treatment. The mean reported percentage of MAA injected uptake by HCC is 32 %, even exceeding 90 % in large and highly vascularized tumors [27]. Tumor-to-non-tumor uptake ratios are usually high, with a mean of 7.2 [27].

For metastases, the clinical situation differs, manifesting as multifocal disease with generally smaller lesions in patients who have received limited or no previous treatment, with variable vascularization levels. In one study by Van de Wiele, the mean MAA incorporated by lesions was only 1.5 % [19]. The tumor-to-non-tumor ratio proved to be typically lower, with a mean value of only 1.7 reported in one study [28].

### Tumor size

As the risk of reflux is reportedly higher in small tumors [23], MAA and microsphere distribution may be discordant in this context.

### Prior therapy

Prior therapy may induce arterial disorders and weaknesses. This is especially the case for patients who have received chemoembolization or antiangiogenic drugs. In this context, the reproducibility of the two angiographic procedures, the first being diagnostic and the second therapeutic, may be impaired due to arterial microlesions induced by the first angiography. Studies have already described high discordance between MAA and microsphere distribution in this context [29].

### The parameters of MAA injection

There are currently no clear recommendations regarding the MAA injection process. Guidelines indicate slow injection is better, ideally lasting 20–30 seconds, into a volume of sufficient size, ideally greater than 5 ml, to best mimic microsphere infusion, with glass microspheres requiring 20 cc, and resin requiring even more.

### Angiographic considerations

The most relevant and widely recognized angiographic consideration is the exact location of MAA and microsphere injection. Both products must be injected into exactly the same position, i.e. the same vessel, the same distance from the tip of the catheter and bifurcations, and the same orientation in the vascular lumen.

Typically, good reproducibility between MAA and microsphere biodistribution is impossible if the MAA is injected into the common hepatic artery and the microspheres into the right or left hepatic artery. This situation can occur in cases of bilateral disease, with one work up involving MAA injection into the common hepatic artery followed by two sequential lobar microsphere injections. In this situation, the liver lobe with the most significant tumor involvement can divert the arterial flow.

This is also the reason why, no matter which artery is selected (right or left hepatic artery), the exact position of the catheter, namely the distance from the tip of the catheter and bifurcations, must be the same. The angle of the catheter may also influence MAA/microsphere distribution.

Nevertheless, even when using the same intended catheter positioning for ^99m^Tc-MAA and microsphere injections, the product distribution can at times differ. This observation was made by Chiesa et al. [12] in 6.8 % of 29 HCC patients treated with glass microspheres, as well as by Jiang et al. [30] in 8.6 % of 81 treatments with resin microspheres. This point underlines the observation that confounding factors potentially influencing particle distribution must be analyzed with great care in order to avoid erroneous conclusions or an overgeneralization of results obtained in a specific situation.

Another key angiographic consideration, which has not been well documented in the scientific literature despite its potentially major impact on MAA and microsphere distribution, is the vasoactive status of the hepatic artery at the time of injection. When performing a dosimetric simulation with MAA, we work under the hypothesis that a diagnostic angiography is identical to a therapeutic one, meaning that the arteries concerned have the same vasoactive status. In reality, this is not necessarily the case, as diagnostic angiographies are typically longer, at times involving aggressive measures like coil embolization that may induce vasospasm.

**Fig. 2:**
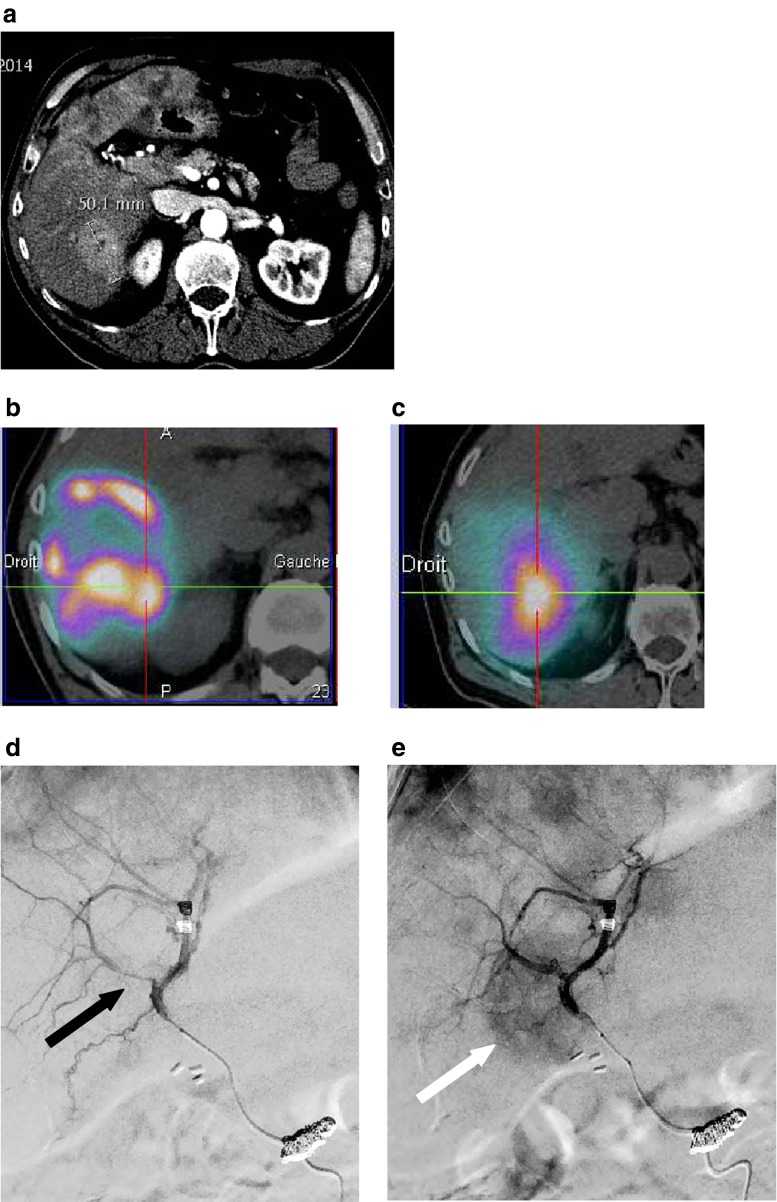
MAA and glass miscrosphere uptake discrepancy related to transient arterial vasospasm. Patient with multifocal neuroendocrine liver metastasis with a 5 cm lesion of segment 6. **a**) Contrast enhanced CT. **b**) Tumor MAA SPECT uptake with a “tumor/non tumor” uptake ratio (T/NT ratio) of 0.77 for segment 6 lesion. **c**) Glass microsphere ^90^Y bremstrahlung SPECT/CT evidencing a better tumor targeting with a T/NT ratio of 3.3 for the same segment 6 lesion. The retrospective analysis of the diagnostic angiography (**d**) and the therapeutic one (**e**) shows an arterial vasospasm (*black arrow*) on the diagnostic angiography with a poor tumoral blush as against no vasospasm and a good tumoral blush (*white arrow*) on the therapeutic one

Using different catheters with varying rigidities can also impact the vasoactive arterial status. Figure 2 provides an example of discordance between MAA and microsphere distribution associated with an arterial spasm that occurred during the MAA injection but not during microsphere injection, owing to differing catheters.

Despite these limitations, MAA-based dosimetry is nonetheless the only available tool for pre-therapy use that may directly impact the treatment schedule. Kao et al. [31] demonstrated a strong correlation between predictive ^99m^Tc-MAA SPECT/CT tumor dose and post-radioembolization yttrium-90 PET dose with resin microspheres. Their experiment was conducted in near-ideal dosimetric conditions, involving well-delineated tumors >2 cm and with a T/NT ratio >2, and thus underlined this dosimetric technique’s great value. Both MAA and PET dosimetry were performed in 23 patients, primarily presenting with HCC (19 cases). The median relative error between both dosimetric evaluations was only 3.8 % (95 % confident interval [CI]: -1.2 % to + 13.2 %), with a trend towards a slight tumor dose overestimation observed with ^99m^Tc-MAA SPECT/CT. This study confirmed the value of MAA as a surrogate to microsphere injection when confounding factors are controlled.

## Lung shunting evaluation and lung dosimetry

### Lung shunting evaluation

The lung shunting fraction (LSF) is defined as the ratio of lung counts to total counts, with extra-hepatic counts considered as gastroduodenal ones. The counts are currently evaluated by means of anterior and posterior planar scan and geometric methods.

In earlier studies [32, 33], LSF was evaluated by means of ^99m^Tc-MAA planar scan. A more extensive study then evaluated 402 patients, 377 with HCC and 25 with liver metastasis [33], revealing higher LSF in the HCC patients (median: 7.6 %; min.: <1 %, max.: 75.4 %) than metastatic (median: 4.7 %; min.: <1 %, max.: 23.9 %). For HCC, lung shunting correlated with tumor size (*p* <0.0001) and tumor vascularity, as assessed by angiography.

### Lung dosimetry

Radiation pneumonitis after ^90^Y-loaded resin microsphere implantation was first described by Leung et al. in 1995 [34]. Five out of the total 80 patients (6.2 %) developed lung injuries, with microspheres present in the lungs on histopathological analysis, informing the radiation pneumonitis diagnosis. The median LSF, evaluated with ^99m^Tc-MAA planar scan, was 23.7 % (min.: 13.1; max.: 45.6) in patients with lung injuries, with an estimated median lung radiation dose of 25 Gy .

The risk of lung injury associated with ^90^Y-loaded glass microsphere injection was evaluated in a group of 58 patients, each receiving a lung dose >30 Gy [35]. In total, 19 (33 %) received a cumulative LD exceeding 50 Gy (mean LD: 75.7 Gy). No clinically relevant lung radiation pneumonitis was observed, with asymptomatic radiological signs of lung disease seen in only 19 %. This observation indicates that the lung dose was overestimated when using planar ^99m^Tc-MAA, with acquisition obtained within 2 hours of administration.

Several factors have been described as having a significant impact on LSF evaluation.

The impact of scatter correction has been analyzed in both phantom [35] and clinical studies [35, 36], with the overall results revealing cases with no scatter correction to be associated with overestimated LSF. In the phantom study, the LSF (calculated on planar images) was 3.2 ± 0.02 % when no scatter correction was used, yet only 0.3 ± 0.01 when it was. Similarly, the mean LSF of three patients was 7.8 ± 2.3 % without scatter correction, yet only 3.6 ± 2.5 with [35]. Another study demonstrated a mean LD overestimation of 40 % when using SPECT/CT without scatter correction in a cohort of 71 patients (Yu et al., 2013).

The delay between ^99m^Tc-MAA injection and image acquisition has also been reported to lead to LSF overestimation [36]. Mean LSF, measured within 1 hour of injection, was 9.3 ± 3.4 %, increasing to 22.1 ± 5.5 % after 5 hours. This increase was more than likely the result of ^99m^Tc-MAA degradation, along with an increase in circulating activities.

Recently, ^99m^Tc-MAA SPECT/CT has been evaluated for its use in LSF calculation. SPECT/CT enables a direct evaluation of lung volume and mass, which can then be used for LD calculation instead of the estimated mass of 1 kg. Yu et al. [37] described an LD calculation based on ^99m^Tc-MAA SPECT/CT (LD_SPECT_) with both scatter and attenuation correction. A lung area located within 2 cm of the diaphragm was excluded in order to avoid misregistration due to breathing. Planar-imaging-based lung dose (LD_plan_) was found to be significantly higher than LD_SPECT,_ with a mean LD_plan_ / LD_SPECT_ ratio of 3.8 ± 4.0 for the 76 evaluated patients. Overestimation of LSF when using planar imaging has been confirmed by Kao et al. [38].

Lastly, due to the difference in size and density of MAA particles compared to microspheres, the accuracy of ^99m^Tc-MAA in predicting microsphere LSF and dosimetry must be fully assessed. One can safely assume that ^99m^Tc-MAA causes LSF overestimation due to the smaller size of the MAA particles. This point was suggested by Salem et al. [35] in their study where no clinically relevant radiation pneumonitis was observed in 53 patients who received a lung dose of over 30 Gy, as evaluated with MAA. This concept was further confirmed by the Elschot et al. study [39], comparing LSF evaluation based on either ^99m^Tc-MAA or ^166^Ho-micospheres. The LD values estimated on the basis of pretreatment diagnostic ^166^Ho-micosphere SPECT/CT (median: 0.02 Gy) were found to be significantly better predictors of the actual LDs, calculated using post-treatment ^166^Ho-micosphere SPECT/CT, compared to doses estimated on the basis of ^99m^Tc-MAA SPECT/CT (median: 2.5 Gy) or planar ^99m^Tc-MAA (median: 5.5 Gy; *p* <0.001).

## Evaluation of the tumoral dose and response

### HCC and tumor dose

With the exception of one study’s negative results, those of the five other available studies were quite concordant, clearly demonstrating there to be a dose/response relationship (Tables 1).

**Table 1:** Studies with MAA based tumor dose evaluation in HCC

	Kucuk [40]	Ho [33]	Kao [41]	Chiesa [12]	Garin [27]	Garin [7]
Product	resin	resin	resin	glass	glass	glass
Dosimetry	NA^a^	MIRD	MIRD	MIRD with BEDs	MIRD	MIRD
Nb patients	19	71	10	52	36	71
Nb lesions	NA	NA	NA	65	58	NA
Lesion size (cm)	NA	NA	NA	5.6	7.1	7.1
Prior therapy (%)	NA	NA	Yes (50) S (NA) CE (NA) Other (NA) No (50)	Yes (28.9) S (15.5) CE (0) Other (13.4) No (71.1)	Yes (42) S (13.8) CE (25) Other (2.7) No (58)	Yes (51) S (22.5) CE (18.3) Other (32.6) No (49)
Number of radioembolization (%)	NA	1 (77.9) 2 to 5 (22.1)	1 (100)	1 (89.6) 2 (10.4)	1 (61) 2 (39)	1 (69) 2 (31)
Response Evaluation	RECIST1.1	WHO	RECIST1.1	EASL	EASL	EASL
Time of evaluation	6 w	NA	NA	3 m	3 m	3 m
Dose/response relationship	NA	YES	Probably^b^	YES	YES	YES
threshold dose (Gy)	NA	225	NA	257	205	205
Impact on survival	NA	NA	NA	NA	YES	YES

^a^ Only a visual evaluation of MMA was done

^b^ All lesion with a TD > 91 Gy responded but all evaluated lesions were responding

Nb = number, NA = non available, S = surgery, CE = chemoembolization, w = week, m = months

The study of Kucuk et al. [40], using resin microspheres in a group of 19 patients, was the only negative outcome study in the HCC setting. The response rate that they recorded at 3 months, defined according to RESIST 1.1 criteria, did not significantly differ between hypoactive or hyperactive lesions, recorded at 40 % and 58 %, respectively (*p* = 0.51). However, this study included no dosimetric evaluation; only a qualitative evaluation of the MAA uptake.

Using the partition model, Ho et al. [33] were the first to report a link between tumor dose and response. In this study, involving 71 HCC patients treated with resin microspheres, the response rate was 37.5 % for lesions with a tumoral dose >225 Gy versus only 10.3 % if the tumoral dose was ≤225 Gy (*p* <0.006). Nevertheless, overall survival did not statistically differ depending on the cumulative tumoral dose (< or ≥300 Gy, *p* = 0.35). In a second study evaluating the resin microsphere method, Kao et al. [41] also reported interesting findings, though this was a preliminary study and involved only eight evaluable patients. Still, all the responding lesions had received a tumoral dose >91 Gy. Owing to all the lesions having responded to treatment, a threshold tumor dose could not be clearly identified in this study.

Chiesa et al. [12] and Mazzaferro et al. [6] conducted studies using glass microspheres, with the former offering preliminary results on 48 patients [12] and the latter offering full results [6] on 52.

A clear dose/response relationship was identified using a dosimetric evaluation based on BEDs at the voxel level. Non-responding lesions had received a median tumor dose of only 199 Gy, compared to 431 Gy for the responding ones (*p* <0.0001) [12]. For response prediction, defining a non-responding lesion with a TD <257 Gy as the true negative and responding lesion with a TD >257 Gy as the true positive, the threshold tumor dose of 257 Gy exhibited a sensitivity of 85 % and specificity of 70 % [12].

In a preliminary study [27], our group also observed a strong dose/response relationship in 36 patients with 58 evaluable lesions of relatively large size (mean size: 7.1 cm). The mean TD was 372.7 ± 142.0 Gy for the 45 responding lesions and only 153.8 ± 80.8 Gy for the 13 non-responding ones (*p* <0.0001). None of the lesions receiving a TD below 205 Gy responded, whereas only five receiving >205 Gy did not respond. This threshold TD of 205 Gy was thus confirmed to be predictive of response, with a sensitivity of 100 % and accuracy of 91 %. According to multivariate analysis, TD was the only parameter that correlated with response (*p* = 0.019). In this study, TD also affected survival. Progression-free survival (PFS) was only 5.2 months when the TD was <205 Gy versus 14 months (*p* = 0.0003) with higher doses. Overall survival (OS) was 9 months when the TD was <205 Gy *versus* 18 months (*p* = 0.0322) with a TD of 205 Gy or higher.

These findings, pertaining to a 205 Gy threshold TD and correlation between TD and survival, were confirmed in a recent study involving 71 patients [7], in which the concept of a personalized dosimetric approach, including treatment intensification when necessary, was also described. The patients who received treatment intensification were administered an ILD ≥150 Gy, contrasting with the 120 ± 20 Gy delivered in the classical approach. In this concept, the HILD was <120 Gy and the LD was typically <30 Gy for one treatment or <50 Gy for several. In total, 38 % of patients received treatment intensification. The response rates were significantly higher with the personalized dosimetric approach than with the standard dosimetric approach, recorded at 86 % versus only 55 %, respectively (*p* = 0.01).

This intensification concept appears to be of particular value for PVT patients. Personalized dosimetry, with treatment intensification where necessary, has been described in a study involving 41 PVT cases [42]. In this trial, 37 % of patients received treatment intensification. A high response rate of 85 % was achieved without causing any increase in liver Grade ≥ III permanent toxicity (6 % versus 12 % in the non-boosted patients, ns). The TD was found to exhibit a highly significant impact on OS, which was 4.3 months (3.7-5 months) versus 18.2 months (8.5–28.7 months) for patients with a TD below 205 Gy or over 205 Gy, respectively (*p* = 0.005). Those with a TD ≥205 Gy and good PVT targeting (n = 36) exhibited an OS of 20.9 months. The objective median OS was not reached, though it was longer than 24.5 months and significantly longer (*p* = 0.0493) for the five patients who underwent lobar hepatectomy.

### Metastatic disease and tumor dose

Several studies have produced disappointing results in this context, yet there are some methodological concerns that could account for this (Table 2). For example, only MAA uptake qualitative evaluation was reported on some occasions, with no mention of type of dosimetric approach [43, 44]. Also, the morphological response evaluation used was at times inappropriate, not including necrosis or hypervascularization evaluation [43, 44], and the means of evaluating delay of response was also insufficient, consisting of 4–6 weeks instead of 3 months [19]. Five studies produced disappointing results, four evaluating resin microspheres and one glass microspheres.

**Table 2:** Studies with MAA based tumor dose evaluation in metastatic disease

	Knesaurek [45]	Wondergem [23]	Dhabuwala [44]	Ulrich [43]	Van de Wiele [19]	Flamen [28]	Lam [48]
Product	resin	resin	resin	resin	glass	resin	resin
Dosimetry	NA ^a^	NA ^b^	NA ^c^	MIRD	MIRD	Monte carlo	MIRD
Nb patients	20	31	58	66	13	8	25
Nb lesions	NA	225 ^b^	NA	435	91	39	NA
Lesion size (cm)	NA	NA	NA	3.4	3.5	5.7	NA
Prior therapy (%)	NA	NA	NA	YES (chemo) (100)	YES (chemo) (100)	YES (chemo) (100)	YES (chemo) (100)
Concomitant Chemotherapy	NA	NA	NA	NA	NA	YES	NA
Number of radioembolization (%)	NA	NA	1 (100)	NA	1 (84.6) 2 (15.4)	NA	1 (100)
Response Evaluation	NA	NA	WHO	RECIST1.1	FDG PET	FDG PET	RECIST1.1
Time of evaluation	NA	NA	2 m	3 m	4-5 weeks	6 weeks	3 m
Dose/response relationship	NA	NA	NA	NA	NA	YES	YES
threshold dose (Gy)	NA	NA	NA	NA	NA	NA	44.2
Impact on survival	NA	NA	NA	NA	NA	NA	YES

^a^ visual assessment of MAA and microspheres in tumors

^b^ quantification of MAA and microspheres in hepatic segment

^c^ visual evaluation of MAA uptake

Nb = number, NA = non available, Chemo = chemotherapy, m = months

Knesaureck et al. [45] reported a comparative study of MAA and resin microsphere uptake in a group of 20 patients (lesion size not available). A strong correlation was demonstrated for some patients, while others showed poor correlation, with Spearman’s rank values of between 0.451 and 0.818.

Wondergem et al. [23], also comparing MAA and resin uptake in 225 hepatic segments, reported disappointing results in 31 patients, primarily involving metastasis. Differences of >10 %, >20 %, and >30 % in the mean activity per milliliter was found in 68 %, 43 %, and 32 % of the 225 segments analyzed, respectively. Tumor burden significantly influenced these differences, with smaller discrepancies observed for segment involvement >25 %.

The 2005 Dhabuwala et al. [44] study reported no correlation between qualitative MAA uptake and response in 58 patients treated by resin microspheres. Furthermore, CT response rates at 3 months did not, in fact, differ between patients with high MAA uptake (n = 37) and those with equivocal or low MAA uptake (n = 21). Nevertheless, tumor response was only evaluated based on lesion size changes. In other studies, the MAA and microsphere injections were not performed in comparable situations, with angiotensin II injected only prior to microsphere administration. Due to its recognized capacity to increase tumoral vascularization [46, 47], MAA was unable to accurately predict microsphere biodistribution in this study.

In their large study involving 66 patients and 435 lesions, Ulrich et al. [43] evaluated the use of resin microspheres and also found no correlation between MAA qualitative uptake and response. The mean tumoral size was relatively small (3.4 cm), with response evaluated solely using lesion size changes. At 3 months, 290 lesions were evaluable according to RECIST 1.1 criteria. In total, 22 % of the responding lesions exhibited low MAA uptake and 21.7 % of the non-responding lesions high MAA uptake.

Van de Wiele et al. [19] conducted another study, this time using glass microspheres, and also found there to be no correlation between the accuracy of MAA quantification and response, as the responding lesions exhibited a mean microsphere activity of 1.95 MBq/cc compared to 1.90 MBq/cc for non-responding ones (*p* = 0.92). However, response was evaluated very early in their study, namely at 4–5 weeks, with the mean lesion size being quite small (3.5 cm) and MAA uptake very low, with a mean uptake of only 1.5 % per lesion. This last finding clearly demonstrates the significantly different vascular behavior of metastasis in comparison with HCC, given that the mean reported MAA uptake in HCC was 32.8 % [27].

Two studies published positive data on this issue.

Flamen et al. [28] conducted a preliminary study evaluating resin microspheres involving eight patients and 39 lesions with a mean size of 5.7 cm. They reported a strong correlation between the tumoral absorbed dose, evaluated on ^99m^Tc-MAA SPECT imaging, and the FDG PET response to ^90^Y-resin microspheres in colorectal metastatic disease using an appropriate dosimetric approach, namely the Monte Carlo simulation. The median simulated absorbed dose was 20 Gy for the 20 poorest responding lesions and 46 Gy for the 19 responding ones (*p* <0.001).

More recently, Lam et al. [48] reported very interesting results involving 25 metastatic patients treated by resin microspheres. In order to avoid bias, only those with identical MAA and microsphere injection sites, as well as no injection failures, were analyzed, with response evaluated at 3 months according to the RECIST 1.1 criteria. Their chosen approach was original, based on both MAA scintigraphy and ^99m^Tc-sulfur colloid (SC) liver scintigraphy for the segmentation between tumor and surrounding liver tissue. Furthermore, the authors used and tested different fixed thresholds for both MAA and SC volume delineation. This consisted of tumor and healthy liver segmentation, with the aim of suppressing manual segmentation, which would be of particular interest in cases of multifocal disease, just as that encountered in metastatic ones. Their dosimetric approach was based on the MIRD concept. The mean TD was 82.7 ± 23.9 Gy for responders *versus* 31.0 ± 10.9 Gy for non-responder*p* (p <0.001). A threshold tumoral dose of 44.2 Gy correlated with response (*p* <0.001). The authors made a very interesting observation that the TD exhibited a clinical impact on survival. The median OS of patients who received a TD of over 55 Gy was 32.8 months, whereas those who received less than 55 Gy had a median survival of only 7.2 months (*p* <0.05). The TD was the only parameter that correlated with survival (*p* <0.01).

## Evaluation of healthy injected liver dose (HILD) and toxicity (Table 3)

**Table 3:** Studies with MAA based healthy injected liver dose evaluation and toxixity

	Youn [44]	Garin [7]	Chiesa [12]	Gulec [49]	Sangro [50]	Lam [48]
Number of patients	41	71	52	40	45	25
Product	glass	glass	glass	resin	resin	resin
Indication	HCC	HCC	HCC	Metastases : 87.5 % HCC : 12.5 %	Primary : 28.9 % Metastases : 71.1 %	metastases
Approach	lobar	lobar	lobar	Whole liver	Whole liver : 73.3 % Lobar : 26.7 %	Whole liver
Cirrhosis and severity	Yes	Yes Child A: 94 % Child B: 6 %	Yes Child A: 83 % Child B: 17 %	no	no	no
Prior therapy (%)	NA	Yes (51) S (22.5) CE (18.3) Other (32.6) No (49)	Yes (28.9) S (15.5) CE (0) Other (13.4) No (71.1)	Yes (100) chemo	Yes (73.3) chemo No (26.7)	Yes (100) chemo
Concomitant therapy	no	no	no	no	29.9 %	no
Definition of toxicity	CTCAE V3	CTCAE V3	Liver decompensation	Liver failure	REILD	CTCAE V4
Severity	G3	G3 and permanent	any	Clinically relevant	any	50 % increase of ALT, AST and ALP
HIL D evaluation	NA	Yes	NA	Yes	Yes	Yes
Correlation HILD and Toxicity	NA	NA	NA	No, but no related tocicity	Yes, but only for whole liver injection	Yes
Threshold HILD	NA	NA	NA	NA	36 Gy, p = 0.02	25.4 Gy, p < 0.01
Other dosimetric parameter correlated with toxicity	Cumulative lobe dose > 390 Gy, *p* < 0.005	HILD ≥ 120 Gy + HR < 30 %, *p* < 0.0001	NTCP, Whole liver dose > 66 Gy, CP of 33 %	HILD till 99Gy is well tolerated	NA	NA

HILD = healthy injected liver dose

HR = hepatic reserve (% of non irradiated liver), NTCP = non tumoral complication probability, CP = complication probability, ALT = alanine aminotransferase, AST = aspartate aminotransferase, ALP = alkaline phosphatise, S = surgery, CE = chemoembolization, Chemo = chemotherapy

### Toxicity evaluation

When discussing liver toxicity, the three following factors should be carefully considered: its imputability, severity, and reversibility.

The imputability of a therapeutic agent in the event of side effects is not always easy to demonstrate. This is typically true for radioembolization, where imputability rules have yet to be defined, since this context may involve a great number of confounding factors, such as tumor progression, underlying liver disease and severity, as well as previous or concomitant chemotherapy.

The severity of an adverse event is usually graded using the Common Terminology Criteria for Adverse Events (CTCAE). For each study, the version used must thus be specified. In cases involving cirrhosis, significant liver toxicities are often encountered at baseline prior to therapy, and only an increase in the toxicity score should thus be reported as an adverse event potentially caused by treatment.

The third point that has been fairly widely discussed is the potential reversibility of the toxicity. The reversibility of toxicities is not evaluated in the CTCAE. In cases of liver toxicity, reversibility is common owing to liver tissue’s ability to regenerate. As a result, severe but transient toxicities are not necessarily limiting or life threatening. For example, when lymphoma is treated by polychemotherapy, transient Grade 3 or 4 bone marrow toxicity almost always occurs, and polychemotherapy is still the standard of care for lymphoma patients.

### Radiation-induced hepatic toxicity syndromes

Known to be associated with external beam radiotherapy, the most common syndrome of liver toxicity is radiation-induced liver damage, or RILD, first described many years ago [51]. This syndrome is secondary to damage to the centrilobular vein and is commonly associated with ascites without jaundice and elevated alkaline phosphatase, or less frequently, transaminase enzymes.

The typical liver toxicity syndrome associated with radioembolization is radioembolization-induced liver damage, or REILD, recently described for the first time by Sangro et al. [50]. REILD is typically associated with ascites, jaundice, bilirubin elevation, a variable elevation of γGT and alkaline phosphatase, and virtually no change in transaminase enzymes.

The exact toxicity syndrome can be more difficult to assess for patients undergoing chemotherapy or chemotherapy-radioembolization combined therapy prior to treatment, as chemotherapeutic agents can also cause hepatic toxicities, such as hepatic failure, cholestasis, or veno-occlusive disease. The precise underlying cause of toxicity in these contexts, either attributed to chemotherapy or radioembolization, is impossible to define, and combined toxicity can occur in cases involving combined treatments.

Here, again, several confounding factors may be encountered, such as the presence and severity of underlying liver disease, previous therapy, concomitant therapy (especially for metastasis), definition of toxicity, various dosimetric approaches, different products, as well as volume of irradiated liver and non-irradiated liver (hepatic reserve).

This last factor, the hepatic reserve, is of great significance. One study reported a Grade 3/4 liver toxicity rate of 82 % in a whole-liver approach, compared to only 12 % (*p* <0.05) in a group of patients without cirrhosis treated by resin microspheres [52]. In another study, liver toxicities were observed solely in cases involving whole-liver injection, but never in those consisting of lobar injection [50].

On the other hand, a hyper-selective (segmental) approach can be taken using high doses with low toxicity profile. The concept of radiation segmentectomy was first described by Riaz et al. [53], using a high mean dose in the treated segment (521 Gy; no HILD evaluation), who reported only 9 % exhibiting Grade 3/4 biochemical toxicities [53]. When comparing this with large-scale hepatectomy, theoretically no acute liver dysfunction should occur, regardless of the irradiated liver dose (or HILD), provided that the hepatic reserve is sufficient.

The last point that should be remembered is the delay between toxicity occurrence and therapy, especially for patients with cirrhosis, where the chronic liver disease can worsen regardless of the treatment administered, if any at all. With radiation therapy, it is well recognized that toxicity can be acute or delayed. In general, however, only acute toxicity is reported with the use of drugs, beginning in the first month of initiation. This is despite the known possibility of delayed toxicities, which, though less common than the toxicities frequently reported in association with radiation therapy, are still a considerable risk. This point is of great interest when comparing the toxicities of radioembolization to those of systemic drugs, such as sorafenib or chemotherapy.

## Several studies have analyzed liver dosimetry and toxicity in HCC, all using glass microspheres

### Liver toxicity frequency in HCC and predisposing factors

The frequency of liver toxicity, regardless of its severity, can reach as high as 96 % in terms of transaminase levels in patients treated with glass microspheres, as shown in a whole-liver approach in the Dancey et al. study [54]. In the same study, however, severe transaminase toxicities occurred in only 23 % of the patients, underlying the need for an accurate severity scoring.

In the Salem et al. study, involving the largest HCC patient cohort, the rate of severe liver toxicities with glass microspheres was found to be quite low, with Grade 3/4 bilirubin toxicity observed in 19 % of 291 patients [3], though this was a conservative analysis, since patients with pre-existing laboratory toxicities were counted as toxicities at follow-up, even if there was no change in grade. No clinically relevant hepatic toxicity was reported by Hilgard et al. in their cohort of 108 patients [5]. Mazzafero et al. reported clinically detectable ascites in 7.7 %, altered bilirubin (≥G3) in 13.5 %, and alkaline phosphatase elevation (≥G3) in 11.5 % of their patients at 3-month follow-up. The liver decompensation rate at 3 months was 23.1 %, defined as the occurrence of any of the following conditions: clinically relevant ascites, total bilirubin >3 mg/dL, hepatic encephalopathy, prothrombin time international normalized ratio >2.2 or variceal hemorrhage [6]. In this study, the definition of liver toxicity was also conservative, given that a clinically relevant ascites level could be classed as CTCAE V3 Grade 2, dictating that only medical treatment was required, or Grade 3, indicating the need for ascites puncture. Clinically relevant ascites can be transient, as reported elsewhere in the literature.

Lastly, permanent severe Grade ≥3 liver toxicities (CTCAE V3) were reported in 8.4 % of a 71-patient cohort [7].

Predisposing factors besides dosimetry, such as the tumor type (infiltrative or not), tumor volume (>70 %), elevated basal transaminases (≥5 N), elevated basal bilirubin (≥2 mg/dL or 34mmmol/L) or a combination of tumor volume ≥50 % with albumin level <30 G/L, [55] have already been described as having an impact on toxicity. In this study, the risk of major liver toxicity related mortality was 18 % for patients presenting with at least one of these five prognostic factors, in comparison with 0 % for those exhibiting none. Underlying severe biliary disease and PVT without MAA targeting have also been reported to be predisposing factors of severe permanent liver toxicity on multivariate analysis [7].

### Liver toxicity and healthy injected liver dose evaluation in HCC (Table 3)

Young et al. [49] were the first to report a statistically significant difference in median cumulative dose to the injected lobe (ILD) between patients with or without toxicity in a group of 20 Okuda 1-classified HCC cases. An ILD of 222 Gy had been administered to the patients with no toxicity, compared to 390 Gy to those with (*p* <0.005). The reported toxicities were only biological in nature and all were ≥ G3 (CTCAE V3). For patients with liver toxicities, the median ILD was significantly higher in the Okuda 1-classified patients than the Okuda 2 ones (390 Gy and 199 Gy, respectively, *p* <0.05). These findings underline the impact of the clinical baseline status on liver tolerance. No evaluation of the healthy injected liver lobe dose (HILD) was performed in this study.

The HILD was recently evaluated in a study [7], where the mean dosimetric evaluation was performed as standard using the MIRD approach on a cohort of 71 carefully selected patients, 94.4 % of whom had a Child Pugh score of A. The injected activity, ILD, HILD, and hepatic reserve did not correlate with severe (CTCAE V3, G ≥3) clinical permanent liver toxicity. The absence of correlation between the HILD and hepatic toxicity can be accounted for by the amount of hepatic reserve. Only the association of a HILD >100 Gy (and >120 Gy) with a hepatic reserve <30 % correlated with severe permanent liver toxicity on univariate analysis. On multivariate analysis, only the association of a HILD >120 Gy with a hepatic reserve <30 % remained correlated with severe permanent liver toxicity. A liver toxicity score has since been proposed, taking into account several parameters, including the association of a HILD >120 Gy with a hepatic reserve <30 % (Table 4). A score ≥3 was predictive of severe permanent liver toxicity with a sensitivity of 83 % and overall accuracy of 97 % [7].

**Table 4:** Liver Toxicity Score (LTS)

Variables	score
HILD > 120 Gy and hepatic reserve <30 %.	0 or 3
Main PVT without MAA targeting	0 or 3
Severe underlying biliary disease	0 or 3
ALT level >5 N	0 or 1
Bilirubin level > 35 μmol/mL	0 or 1
Tumoral involvement >70 %	0 or 1
Child B	0 or 1
Previously treated patients	0 or 1
Patient LTS	0 to 14

For each variable: 0 point is attributed if it is absent and 1 or 3 points are attributed if present

The patient score is obtained by adding the number of points attributed to each variable

The score is considered positive (predictive of liver toxicity) if its value is ≥ 3

The LTS was designed to predict, prior therapy, the risk of liver failure. A score ≥ 3 was predictive of severe permanent liver toxicity with a sensitivity of 83 %, i.e., only one toxicity missed, and overall accuracy of 97 %.

In a recent study, Chiesa et al. [12] calculated the global dose to the healthy liver, including to the irradiated and non-irradiated parenchyma. This parameter is still currently used in practice, particularly valuable in external beam radiation therapy, as both lobes typically receive a varying amount of radiation due to the use of multiple irradiation beams. With the use of this parameter, normal tissue complication probability (NTCP) can then be calculated. Chiesa et al. found that the risk of liver decompensation (as defined by Mazzaferro et al. [6]) was 0 %, 14 %, 40 %, and 67 % for global healthy liver dose intervals of 0–35 Gy, 35–70 Gy, 70–105 Gy, and 105–140 Gy, respectively [24]. The authors suggested fixing a limit of 70 Gy for the global healthy liver dose, a concept validated in one particular case where, corresponding to an NTCP of approximately 15 % of liver decompensation, glass microspheres were implanted 3.75 days after the calibration date, corresponding to a defined specific activity.

The use of this NTCP model in radioembolization must be clinically assessed, especially in cases involving unilobar injection, as it has been validated for external beam radiotherapy in a specific configuration of irradiation, especially in terms of dose rate, that differs from that encountered in radioembolization. For example, for a patient with unilobar injection, namely where one lobe receives no irradiation, who exhibits a hepatic reserve of 50 %, it is more than likely that no matter what amount of total healthy liver dose is received, the treatment will be well tolerated due to this high hepatic reserve, especially in comparison to surgery.

### For metastatic disease, studies analyzing liver toxicity and dosimetry are extremely scarce (Table 3)

All these studies were performed using resin microspheres. Cases of metastatic disease present more difficulties in analyzing toxicity and dosimetry, as most patients have typically received either limited or no treatment, or a combination of chemotherapy and radioembolization.

### Liver toxicity frequency in metastatic disease and predisposing factors

In a multicenter international collaboration, Kennedy et al. [22] reported the outcome of 680 treatments with resin microspheres for non-resectable hepatic tumors, primarily metastases (86 %). This is the largest cohort of its kind described to date, with chemotherapy used prior to treatment in 66 % of cases.

The authors evaluated the risk of liver toxicity, using the term REILD. REILD-related death was described in 28 of 680 treatments (4 %), with 21 occurring in one particular center that used the empirical method. HILD was not evaluated in this study, though several factors were identified as correlating with RIELD, such as activity delivered (*p* <0.0001) or prescribed (*p* <0.0001), percentage of empiric activity delivered (*p* <0.0001), number of prior liver treatments (*p* <0.0008), medical center (*p* <0.0001), and treatment of the right lobe (*p* = 0.0008).

In the Sangro et al. study, REILD was diagnosed in 20 % of the patients (i.e., ten cases out of 45) who received resin microspheres [50]. Three cases were severe and resulted in death. REILD was only observed in patients who had received prior chemotherapy and in those undergoing whole-liver treatment (*vs.* lobar). In addition, the authors identified several other predisposing factors for liver toxicity in patients receiving whole-liver radioembolization, including HCC diagnosis (*p* = 0.002), flow redistribution during angiographic procedure (*p* = 0.004), and activity administered relative to total-liver volume (*p* = 0.003).

### Liver toxicity and healthy injected liver dose evaluation in metastatic disease

Using the partition model, Gulec et al. [56] were the first to evaluate HILD, treating 40 patients who were primarily metastatic disease cases with resin microspheres in single whole-liver administration. The mean HILD was 17.2 Gy (min.: 0.7; max.: 99.5 Gy). No liver toxicity was observed in this study and the authors concluded that the healthy liver tissue tolerated doses of up to 99.5 Gy.

The mean HILD was also evaluated using the partition model in the Sangro et al. study [50], involving 31 patients receiving whole-liver radioembolization. This value differed significantly (*p* = 0.02) between the REILD (36.7 Gy) and non-REILD groups (25.7 Gy).

Lam et al. recently published interesting results for their study using a dosimetric approach based on MIRD and a fusion MAA SPECT/SC SPECT for the segmentation between tumors and healthy liver tissue [48]. The trial involved 25 patients with colorectal metastases treated with a lobar approach. They all had received heavy previous treatment and only three underwent systemic therapy following radioembolization. The authors reported a correlation (r = 0.38-0.69; *p* <0.01) between the HILD and change in serum liver enzymes (aspartate aminotransferase [AST], alanine transaminase [ALT], and alkaline phosphatase). The HILD was found to be an accurate predictor of broad biochemical toxicity, defined as a 50 % increase in each of the three liver enzymes, given that all were increased at doses ≥24.5 Gy. No toxicity higher than CTCAE V4 Grade 1 was observed for these enzymes.

### Post-therapeutic bremsstrahlung or PET dosimetry

Post-therapeutic dosimetry is believed to be more accurate than MAA dosimetry due to its capacity to evaluate the true dose corresponding to the real distribution of the therapeutic agent, rather than the estimated dose based on the distribution of a surrogate measure. Post-therapeutic dosimetry is thus required to define, as precisely as possible, the tumoral threshold doses and liver maximal tolerated dose.

Post-therapeutic dosimetry cannot be used for a selected patient to adapt the treatment schedule, such as in an attempt to optimize the activity that has to be injected in order to reach the tumoricidal dose while not exceeding the maximal tolerated liver dose. However, post-therapeutic dosimetric evaluation does provide the effectively received doses and thus confirms the MAA findings.

The most relevant study assessing post-therapeutic dosimetry was published by Strigari et al. [14]. The authors investigated dose distribution using post-treatment (3-dimensional) bremsstrahlung activity distribution and Monte Carlo dose voxel kernel calculations. The mean doses to the tumors and healthy injected liver were calculated for 73 HCC patients treated with resin microspheres. The toxicity was graded using the CTCAE V4. The median HILD was 36 Gy (range: 6–78 Gy), and Grade ≥2 (G2), ≥3 (G3), and ≥4 (G4) liver toxicities were observed in 32 % (23/73), 21 % (15), and 11 % (8) of patients, respectively. A healthy injected liver dose of 52 Gy (95 % CI: 44–61 Gy) was identified in a whole-liver injection, providing a 50 % probability of ≥ G2 liver toxicity in this patient group.

Post-therapeutic PET dosimetry has also been described as technically possible due to a small probability of positron emission in the ^90^Y decay [57]. Several studies have provided evidence that ^90^Y PET is superior to ^90^Y bremsstrahlung for tumors and non-target tissue detection (especially for the stomach) and dosimetry [58].

In one study, ^90^Y PET dosimetry was available for four patients with gastroduodenal resin microsphere uptake [30]. One patient with a mean gastric dose of 18 Gy remained asymptomatic, while the remaining three, having received gastric or duodenal doses ≥49 Gy, suffered from gastritis or duodenitis.

### Perspectives

Prospective trials with both MAA and post-therapeutic dosimetry must now be performed to confirm these preliminary results observed retrospectively, and in order to obtain the most valuable evaluation of tumoral threshold dose and maximal liver tolerated dose. Such an evaluation should take into account all potential confounding factors, such as indication, tumor size, product used, and so on, to avoid bias and erroneous conclusions.

Due to the concordant results found in HCC patients, a personalized dosimetric approach using MAA should be implemented in the near future. For metastatic disease, more studies must be carried out before we are able to define a more personalized approach, as the results are more contradictory in this context.

## Conclusion

^99m^Tc-MAA SPECT/CT-based dosimetry is of major interest in radioembolization conducted with radiolabeled microspheres. This method enables accurate patient selection, excluding patients with high risk of lung shunting from therapy, while the quantification of lung shunting can be further optimized. ^99m^Tc-MAA SPECT/CT-based dosimetry has brought to light a clear dose/response relationship in HCC patients in various independent studies. There have been contradictory results published in the metastatic disease context, with the more recent results providing the most value. A correlation between tumor dose and overall survival has also been demonstrated. The possibility of achieving a personalized dosimetric technique has now been advanced. Calculating the healthy injected liver dose and predicting toxicity are still highly challenging processes, despite several studies having demonstrated a high correlation between liver toxicities and HILD or other parameters related to whole-liver dose, as well as between liver toxicities and a combination of HILD and the hepatic reserve. If such a personalized approach becomes possible on account of these advances, it could have a significant clinical impact. ^99m^Tc-MAA SPECT/CT must be more extensively studied and is certain to benefit from future improvements, such as dose volume histogram generation, BEDs, and eventually, more complex approaches like the Monte Carlo simulation and dose kernel point evaluation.

## References

[1] Gray B, Van Hazel G, Hope M, Burton M, Moroz P, Anderson J (2001). Randomised trial of SIR-Spheres plus chemotherapy vs. chemotherapy alone for treating patients with liver metastases from primary large bowel cancer. Ann Oncol.

[2] Hendlisz A, Van den Eynde M, Peeters M, Maleux G, Lambert B, Jaarke Vannoote J (2010). Phase III Trial Comparing Protracted Intravenous Fluorouracil Infusion Alone or With Yttrium-90 Resin Microspheres Radioembolization for Liver-Limited Metastatic Colorectal Cancer Refractory to Standard Chemotherapy. J Clin Oncol.

[3] Salem R, Lewandowski RJ, Mulcahy MF (2010). Radioembolization for hepatocellular carcinoma using Yttrium-90 microspheres: a comprehensive report of long-term outcomes. Gastroenterology.

[4] Sangro B, Carpanese L, Cianni R, Golfieri R, Gasparini D, Ezziddin S (2011). European Network on Radioembolization with Yttrium-90 Resin Microspheres (ENRY). Survival after yttrium-90 resin microsphere radioembolization of hepatocellular carcinoma across Barcelona clinic liver cancer stages: a European evaluation. Hepatology.

[5] Hilgard P, Hamami M, Fouly AE, Scherag A, Müller S, Ertle (2010). Radioembolization with yttrium-90 glass microspheres in hepatocellular carcinoma: European experience on safety and long-term survival. Hepatology.

[6] Mazzaferro V, Sposito C, Bhoori S, Romito R, Chiesa C, Morosi C (2013). Yttrium(90) radioembolization for intermediate-advanced hepatocarcinoma: A phase II study. Hepatology.

[7] Garin E, Lenoir L, Edeline J, Laffont S, Mesbah H, Porée P (2013). Boosted selective internal radiation therapy with ^90^Y-loaded glass microspheres (B-SIRT) for hepatocellular carcinoma patients: a new personalized promising concept. Eur J Nucl Med Mol Imaging.

[8] Raoul JL, Guyader D, Bretagne JF, Duvauferrier R, Bourguet P, Bekhechi D (1994). Randomized controlled trial for hepatocellular carcinoma with portal vein thrombosis: intra-arterial iodine-131-iodized oil versus medical support. J Nucl Med.

[9] Lau WY, Kennedy AS, Kim YH, Lai HK, Lee RC, Leung TW (2012). Patient selection and activity planning guide for selective internal radiotherapy with yttrium-90 resin microspheres. Int J Radiat Oncol Biol Phys.

[10] Sangro B, Iñarrairaegui M, Bilbao JI (2012). Radioembolization for hepatocellular carcinoma. J Hepatol.

[11] Garin E, Bourguet P. Radiolipiodol of liver tumors. Use of labelled lipiodol in the treatment of hepatic tumors. In “Nuclear Medicine in Clinical Diagnosis and Treatment”, 3rd edition, vol 1, Ell and Gambhir, Churchill Livingstone, Elsevier Science Ltd., 2004; 473-483.

[12] Chiesa C, Maccauro M, Romito R, Spreafico C, Pellizzari S, Negri A (2011). Need, feasibility and convenience of dosimetric treatment planning in liver selective internal radiation therapy with 90Y microspheres: the experience of the National Tumor Institute of Milan. Q J Nucl Med Mol Imaging.

[13] Garin E, Lenoir L, Rolland Y, Laffont S, Pracht M, Mesbah H (2011). Effectiveness of quantitative MAA SPECT/CT for the definition of vascularized hepatic volume and dosimetric approach: phantom validation and clinical preliminary results in patients with complex hepatic vascularization treated with yttrium-90-labeled microspheres. Nucl Med Commun.

[14] Strigari L, Sciuto R, Rea S, Carpanese L, Pizzi G, Soriani A (2010). Efficacy and toxicity related to treatment of hepatocellular carcinoma with 90Y-SIR spheres: radiobiologic considerations. J Nucl Med.

[15] Cremonesi M, Ferrari M, Bartolomei M, Orsi F, Bonomo G, Aricò D (2008). Radioembolisation with 90Y-microspheres: dosimetric and radiobiological investigation for multi-cycle treatment. Eur J Nucl Med Mol Imaging.

[16] Ho S, Lau WY, Leung TWT, Chan M, Ngar YK, Johnson PJ (1996). Partition model for estimating radiation doses from yttrium-90 microspheres in treating hepatic tumours. Eur J Nucl Med.

[17] Sirtex Medical. //www.sirtex.com/us/clinicians/package-insert/ 2005.

[18] Salem R, Lewandowski RJ, Gates VL, Nutting CW, Murthy R, Rose SC (2011). Technology Assessment Committee; Interventional Oncology Task Force of the Society of Interventional Radiology. Research reporting standards for radioembolization of hepatic malignancies. J Vasc Interv Radiol.

[19] Van de Wiele C, Maes A, Brugman E, D’Asseler Y, De Spiegeleer B, Mees G (2012). SIRT of liver metastases: physiological and pathophysiological considerations. Eur J Nucl Med Mol Imaging.

[20] De Gersem R, Maleux G, Vanbilloen H, Baete K, Verslype C, Haustermans K (2013). Influence of time delay on the estimated lung shunt fraction on 99mTc-labeled MAA scintigraphy for 90Y microsphere treatment planning. Clin Nucl Med.

[21] Bilbao JI, de Martino A, de Luis E, Díaz-Dorronsoro L, Alonso-Burgos A, Martínez de la Cuesta A (2009). Biocompatibility, inflammatory response, and recannalization characteristics of nonradioactive resin microspheres: histological findings. Cardiovasc Intervent Radiol.

[22] Kennedy AS, McNeillie P, Dezarn WA, Nutting C, Sangro B, Wertman D (2009). Treatment parameters and outcome in 680 treatments of internal radiation with resin 90Y-microspheres for unresectable hepatic tumors. Int J Radiat Oncol Biol Phys.

[23] Wondergem M, Smits ML, Elschot M, de Jong HW, Verkooijen HM, van den Bosch MA (2013). 99mTc-macroaggregated albumin poorly predicts the intrahepatic distribution of 90Y resin microspheres in hepatic radioembolization. J Nucl Med.

[24] Walrand S, Hesse M, Chiesa C, Lhommel R, Jamar F (2014). The Low Hepatic Toxicity per Gray of 90Y Glass Microspheres Is Linked to Their Transport in the Arterial Tree Favoring a Nonuniform Trapping as Observed in Posttherapy PET Imaging. J Nucl Med.

[25] Chiesa C, Mira M, Maccauro M, Romito R, Spreafico C, Sposito C (2012). A dosimetric treatment planning strategy in radioembolization of hepatocarcinoma with 90Y glass microspheres. Q J Nucl Med Mol Imaging.

[26] Garin E (2011). Radioembolisation of hepatocellular carcinoma patients using yttrium-90 labelled microspheres : towards a diffusion of the technique?. Eur J Nucl Med.

[27] Garin E, Lenoir L, Rolland Y, Edeline J, Mesba H, Laffont S (2012). ^99m^Tc-MAA SPECT/CT based dosimetry accurately predicts tumour response and survival in HCC patients treated with ^90^Y-loaded glass microspheres : preliminary results. J Nucl Med.

[28] Flamen P, Vanderlinden B, Delatte P, Ghanem G, Ameye L, Van Den Eynde M (2008). Multimodality imaging can predict the metabolic response of unresectable colorectal liver metastases to radioembolization therapy with Yttrium-90 labeled resin microspheres. Phys Med Biol.

[29] Garin E, Rolland Y, Boucher E, Ardisson V, Laffont S, Boudjema K (2010). First experience of hepatic radioembolization using microspheres labelled with yttrium-90 (TheraSphere®): practical aspects concerning its implementation. Eur J Nucl Med Mol Imaging.

[30] Jiang M, Fischman A, Nowakowski F, Heiba S, Zhang Z, Knesaurek K (2012). Segmental perfusion differences on paired Tc-99 m Macroaggregated Albumin (MAA) hepatic perfusion imaging and Yttrium-90 (Y-90) bremsstrahlung imaging studies in SIR-Sphere radioembolization: associations with Angiography. J Nucl Med Radiat Ther.

[31] Kao YH, Steinberg JD, Tay YS, Lim GK, Yan J, Townsend DW (2013). Post-radioembolization yttrium-90 PET/CT - part 2: dose-response and tumor predictive dosimetry for resin microspheres. EJNMMI Res.

[32] Leung WT, Lau WY, Ho SK, Chan M, Leung NW, Lin J (1994). Measuring lung shunting in hepatocellular carcinoma with intrahepatic-arterial technetium-99m macroaggregated albumin. J Nucl Med.

[33] Ho S, Lau WY, Leung TWT, Chan M, Johnson PJ, Li AKC (1997). Clinical evaluation of the partition model for estimating radiation doses from yttrium-90 microspheres in the treatment of hepatic cancers. Eur J Nucl Med.

[34] Leung TW, Lau WY, Ho SK, Ward SC, Chow JH, Chan MS (1995). Radiation pneumonitis after selective internal radiation treatment with intraarterial 90yttrium-microspheres for inoperable hepatic tumors. Int J Radiat Oncol Biol Phys.

[35] Salem R, Parikh P, Atassi B, Lewandowski RJ, Ryu RK, Sato KT (2008). Incidence of radiation pneumonitis after hepatic intra-arterial radiotherapy with yttrium-90 microspheres assuming uniform lung distribution. Am J Clin Oncol.

[36] O’Doherty J, Scuffham J, Hinton P (2011). The importance of scatter correction for the assessment of lung shunting prior to yttrium-90 radioembolization therapy. Nucl Med Commun.

[37] Yu N, Srinivas SM, Difilippo FP, Shrikanthan S, Levitin A, McLennan G (2013). lung dose calculation with SPECT/CT for 90Yttrium radioembolization of liver cancer. Int J Radiat Oncol Biol Phys.

[38] Kao YH, Magsombol BM, Toh Y, Tay KH, Chow PK, Goh AS (2014). Personalized predictive lung dosimetry by technetium-99m macroaggregated albumin SPECT/CT for yttrium-90 radioembolization. EJNMMI Res.

[39] Elschot M, Nijsen JF, Lam MG, Smits ML, Prince JF, Viergever MA (2014). (99m)Tc-MAA overestimates the absorbed dose to the lungs in radioembolization: a quantitative evaluation in patients treated with (166)Ho-microspheres. Eur J Nucl Med Mol Imaging.

[40] Kucuk ON, Soydal C, Araz M, Ozkan E, Aras G (2013). Evaluation of the response to selective internal radiation therapy in patients with hepatocellular cancer according to pretreatment (99m)Tc-MAA uptake. Clin Nucl Med.

[41] Kao YH, Hock Tan AE, Burgmans MC, Irani FG, Khoo LS, Gong Lo RH (2012). Image-guided personalized predictive dosimetry by artery-specific SPECT/CT partition modeling for safe and effective 90Y radioembolization. J Nucl Med.

[42] Garin E, Rolland Y, Edeline J, Icard N, Lenoir L, Laffont S (2015). Personalized dosimetry and intensification concept with 90Y-loaded glass microsphere radioembolization induce prolonged overall survival in hepatocelluar carcinoma patients with portal vein thrombosis. J Nucl Med.

[43] Ulrich G, Dudeck O, Furth C, Ruf J, Grosser OS, Adolf D (2013). Predictive value of intratumoral 99mTc-macroaggregated albumin uptake in patients with colorectal liver metastases scheduled for radioembolization with 90Y-microspheres. J Nucl Med.

[44] Dhabuwala A, Lamerton P, Stubbs RS (2005). Relationship of 99mtechnetium labelled macroaggregated albumin (99mTc-MAA) uptake by colorectal liver metastases to response following Selective Internal Radiation Therapy (SIRT). BMC Nucl Med.

[45] Knesaurek K, Machac J, Muzinic M, DaCosta M, Zhang Z, Heiba S (2010). Quantitative comparison of yttrium-90 (90Y)-microspheres and technetium-99m (99mTc)-macroaggregated albumin SPECT images for planning 90Y therapy of liver cancer. Technol Cancer Res Treat.

[46] Burton MA, Gray BN, Self GW, Heggie JC, Townsend PS (1985). Manipulation of experimental rat and rabbit liver tumor blood flow with angiotensin II. Cancer Res.

[47] Burton MA, Gray BN, Coletti A (1988). Effect of angiotensin II on blood flow in the transplanted sheep squamous cell carcinoma. Eur J Cancer Clin Oncol.

[48] Lam MG, Goris ML, Iagaru AH, Mittra ES, Louie JD, Sze DY (2013). Prognostic utility of 90Y radioembolization dosimetry based on fusion 99mTc-macroaggregated albumin-99mTc-sulfur colloid SPECT. J Nucl Med.

[49] Young JY, Rhee TK, Atassi B, Gates VL, Kulik L, Mulcahy MF (2007). Radiation dose limits and liver toxicities resulting from multiple yttrium-90 radioembolization treatments for hepatocellular carcinoma. J Vasc Interv Radiol.

[50] Sangro B, Gil-Alzugaray B, Rodriguez J, Sola I, Martinez-Cuesta A, Viudez A (2008). Liver disease induced by radioembolization of liver tumors: description and possible risk factors. Cancer.

[51] Reed GB, Cox AJ (1966). The human liver after radiation injury. A form of veno-occlusive disease. Am J Pathol.

[52] Seidensticker R, Seidensticker M, Damm R, Mohnike K, Schütte K, Malfertheiner P (2012). Hepatic toxicity after radioembolization of the liver using (90)Y-microspheres: sequential lobar versus whole liver approach. Cardiovasc Intervent Radiol.

[53] Riaz A, Gates VL, Atassi B, Lewandowsky R, Mulcahy MF, Ryu R (2011). Radiation segmentectomy: a novel approach to increase safety and efficacy of radioembolization. Int J Radiat Oncol Biol Phys.

[54] Dancey JE, Shepherd FA, Paul K, Sniderman KW, Houle S, Gabrys J (2000). Treatment of non respectable hepatocellular carcinoma with intrahepatic ^90^yttrium microspheres. J Nucl Med.

[55] Goin JE, Salem R, Carr BI, Dancey JE, Soulen MC, Geschwind JF (2005). Treatment of unresectable hepatocellular carcinoma with intrahepatic yttrium 90 microspheres: a risk-stratification analysis. J Vasc Interv Radiol.

[56] Gulec SA, Mesoloras G, Dezarn WA, McNeillie P, Kennedy AS (2007). Safety and efficacy of Y-90 microsphere treatment in patients with primary and metastatic liver cancer: the tumor selectivity of the treatment as a function of tumor to liver flow ratio. J Transl Med.

[57] Gates VL, Esmail AA, Marshall K, Spies S, Salem R (2011). Internal pair production of 90Y permits hepatic localization of microspheres using routine PET: proof of concept. J Nucl Med.

[58] Kao YH, Steinberg JD, Tay YS, Lim GK, Yan J, Townsend DW (2013). Post-radioembolization yttrium-90 PET/CT - part 1: diagnostic reporting. EJNMMI Res.

